# Take Fatigue or Fatigues into Account in Physiotherapy Interventions? A Rapid Scoping Review

**DOI:** 10.1298/ptr.R0038

**Published:** 2025-11-15

**Authors:** Shotaro TACHIBANA, Clémence Kiho BOURGEOIS YOSHIOKA

**Affiliations:** 1 Department of Pediatrics Rehabilitation Medicine l’Escale, Hospices Civils de Lyon (HCL), Bron, France; 2 Trajectoires Team, Lyon Neuroscience Research Center (CRNL), Bron, France; 3 Department of Health Sciences, Medical School, Nagoya City University, Japan

**Keywords:** Fatigue, Physiotherapy, Physical therapist, Rehabilitation

## Abstract

Fatigue is one of the most common symptoms encountered in rehabilitation and during physical therapy interventions. Although this phenomenon is known and experienced by everyone, its assessment is not straightforward. The lack of consensus on its definition, complex etiology, and multidimensional nature means that a large number of outcomes exist and continue to be reviewed. However, it seems essential that its assessment be better defined and standardized to understand the effects of physical therapy. To provide an initial exploratory overview, we conducted a rapid scoping review of the various fatigue assessments used in physiotherapy interventions or performed by physical therapists. A total of 139 articles published between 2020 and July 31, 2025 were included and explored. We found 43 different outcomes used across 46 population groups. While the most well-known chronic conditions such as cancer, multiple sclerosis (MS), and coronavirus disease 2019 (COVID-19) are representative, their assessment methods do not appear to be harmonized. These findings from the study support the idea that fatigue remains a complex phenomenon to assess. However, it appears that the lack of justification for the choice of an outcome prevents a better understanding of the reproducible effects on fatigue in physiotherapy interventions.

## Introduction

Fatigue is a conscious sensation experienced by all individuals. Due to its multidimensional nature, there is currently no universal definition of fatigue. Angelo Mosso, one of the pioneers in the physiological description of fatigue, had already observed the following relationship more than a century earlier: the brain and the muscles alter their function during exercise and that fatigue is predominantly an emotion, part of a complex regulation, whose role is to protect the body from harm^1)^. Although the etiology of fatigue is not yet well understood today, fatigue appears to be a universal phenomenon that can be experienced by any conscious individual. The global prevalence of general fatigue (fatigue lasting <6 months, or fatigue of unspecified duration) is 20.4% in adults, 11.7% in minors, and 42.3% in specific occupations^2)^. In the medical field, one-fifth of family medicine patients present with fatigue, and one-third of adolescents report having fatigue at least 4 days per week^3)^. This observation leads us to an initial reflection. On the one hand, fatigue is a phenomenon already familiar to individuals in their daily lives. On the other hand, the onset of a health problem could cause certain aspects of fatigue to appear or become more pronounced. In other words, the gap between the fatigue already experienced by the individual and the fatigue caused by an illness remains poorly understood. While the causes are diverse and can be physiological and/or psychological in origin, the consequences are often harmful to the individual. Fatigue is one of the most burdensome and disabling symptoms in a multitude of acute and chronic conditions across the lifespan^4)^. Managing fatigue is both a challenge and a key goal in rehabilitation care to improve patients’ quality of life. It is essential to take this into account for rehabilitation follow-up, but the multidimensional nature of fatigue can make it difficult to assess. Appropriate measurement of fatigue allows the effectiveness of an intervention from a clinical perspective, but is also important from a scientific perspective when compared with other studies. Although numerous outcomes exist, including specific ones for certain pathologies, there appears to be no consensus on their use among both clinicians and researchers^5,6)^. Particularly in studies investigating exercise programs performed and/or supervised by physical therapists, fatigue often appears as a primary or secondary outcome measure. A general overview of all the scales used in physiotherapy would contribute to the development of a consensus on fatigue assessment and also provide a conceptual reflection into fatigue assessment itself. For this purpose, our objective is to collect and map the various fatigue-related outcomes used by physical therapists in different areas of intervention. Given the exploratory nature of our study and the heterogeneity of the various parameters we aim to collect, we conducted a rapid scoping review to answer the following 2 questions: What outcomes are used to evaluate fatigue in physiotherapy interventions? And are our fatigue assessments standardized across similar fields?

## Materials and Methods

A scoping review protocol was prepared and registered on the Open Science Framework (osf.io/vcjde), and was guided by established scoping review and rapid review methodologies^7,8)^. This project was conducted over a 14-week timeframe (April 28–August 15, 2025) and was reported according to Preferred Reporting Items for Systematic Reviews and Meta-Analyses extension for scoping reviews (PRISMA-ScR) statement (Appendix 1)^9)^. We provide the search string strategies for each database in Appendix 2.

### Literature search

One researcher (S.T.) conducted a literature search across 6 databases on July 31, 2025, to access a comprehensive list of sources. The search was guided by the JBI Manual for Evidence Synthesis^10)^. Six databases, PubMed (National Library of Medicine), Cochrane Library, CINAHL (EBSCO), Embase (Elsevier), and Web of Science (Clarivate), and LiSSA (a French Health Science Database), were chosen for their inclusion of randomized clinical trials and broad clinical scope. These databases also use controlled vocabularies for physical therapist, rehabilitation, and fatigue (Medical Subject Headings [MeSH], Emtree, CINAHL Subject Headings), which supplement keyword searches for terms appearing in titles, abstracts, and author-selected keywords. Appendix 2 provides the search string strategies for each database. We limited the scope to English-language articles published primarily between 2015 and 2025 to capture current discussion. Publications meeting search criteria were transferred into Rayyan, a web-based systematic review application that was used to assist in screening^11)^.

### Inclusion criteria

Using the PICOS tool, our inclusion criteria were defined as follows:

- Population = Healthy population or people with disease, including children
- Interventions = All types of interventions, directly or remotely, involving a physical therapist
- Comparator = No criteria for comparison (with or without)
- Outcomes = Any scales or questionnaires designed primarily to measure the fatigue experienced by the subject
- Study designs = All study designs, including qualitative studies

### Literature screening

**Fig. 1.**
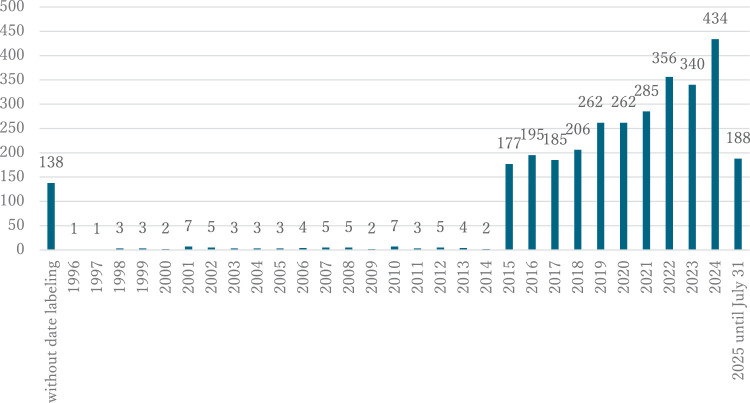
Records found during the screening process per year of publication.

Articles were eligible for inclusion if they were written in English. Although our research was primarily focused on articles published from 2015 onwards, older articles and articles without date labeling were found during the screening process (Fig. 1). First, we eliminated articles published before 2015 and retained only those that used a fatigue outcome in the context of rehabilitation involving physical therapy or a physical therapist. This included articles presenting different types of studies ranging from case studies to qualitative studies. Articles were excluded when fatigue was not explicitly sought in the outcome measures or if it was only reported by a quality of life questionnaire or mentioned as an adverse event. Terms that could be interpreted as synonyms, such as exacerbation or dyspnea, were also excluded to retain articles in which the authors explicitly mentioned fatigue in their work.

**Fig. 2.**
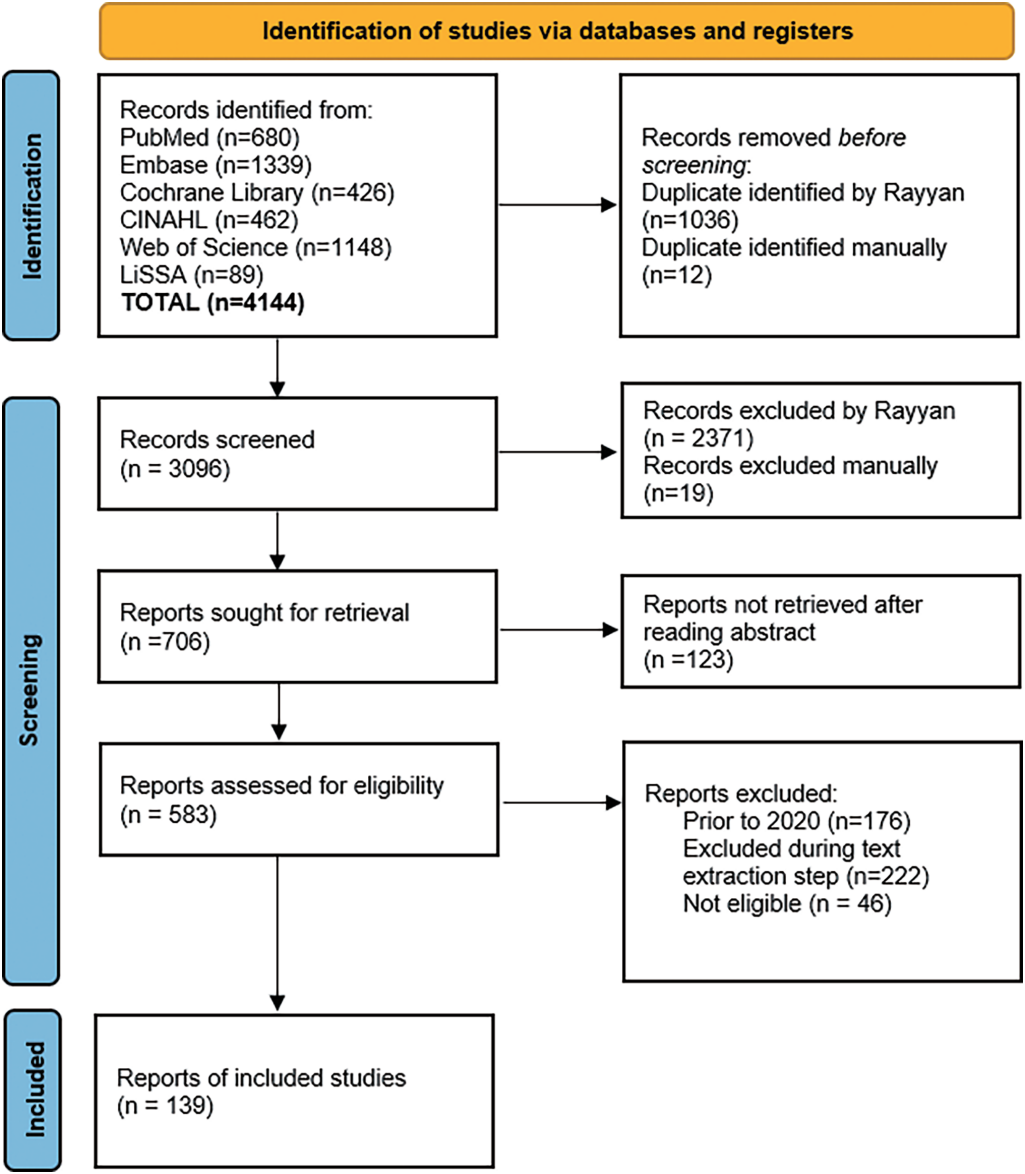
PRISMA diagram showing the results of the literature. PRISMA, Preferred Reporting Items for Systematic Reviews and Meta-Analyses

Literature screening was conducted independently by both authors using Rayyan (Qatar Computing Research Institute, Doha, Qatar), to review all articles and to confirm eligibility at each stage; conflicts were resolved through discussion and consensus. First, titles and abstracts were screened, then the articles were retrieved, and full texts were assessed for eligibility. The screening process was piloted at each stage to clarify eligibility criteria (i.e., refine exclusion criteria). Protocol articles without results, posters, and conference presentations were excluded during this phase because fatigue outcomes were not always specified. Due to the large number of articles meeting eligibility criteria and resource considerations, only studies published in 2020 or later were included in the final set for collecting and mapping the targeted data (Fig. 2).

### Synthesis of results

All articles in the final set were uploaded into the Zotero reference manager (version 7.0.13; Corporation for Digital Scholarship, Vienna, VA, USA). Both authors reread each article and highlighted the following data: population, the criterion(s) used to assess fatigue, and the type of study. The data were entered and classified in a table using Excel software (version 1808; Microsoft, Redmond, WA, USA) to obtain an overview of the different fatigue scales used in different populations. Each author checked the table to validate the final version (Table 1).

**Table 1.** Overview of the different fatigue scales used in different populations

Population	Outcomes for fatigue	Study type	References	Year
Acute ankle sprains	VAS	Longitudinal cohort study	Correia et al.	2021
Acute burn injuries	Hooper’s Index	Interventional study	Page et al.	2025
Amyotrophic lateral sclerosis	FSS	Interventional study	Gonçalves et al.	2022
Amyotrophic lateral sclerosis	MFIS; FSS; NFI-MND	Systematic review	Silva et al.	2024
Amyotrophic lateral sclerosis	FSS; BRS	Systematic review	Souza et al.	2025
Beta thalassemia major	FSS	Case report	Choubisa et al.	2022
Bronchiectasis (adult)	HRQoL	Delphi study	Hamzeh et al.	2024
Cancer	FACT-F; BFI; Fatigue (not specified)	Systematic review	Al-Mhanna et al.	2022
Cancer	EORTC QLQ-C-30	Feasibility study	Christensen et al.	2025
Cancer	FSS	Feasibility study	Denti et al.	2024
Cancer	MFI; EORTC QLQ-C30; PROMs	Systematic review	Gauchez et al.	2024
Cancer	PipFS; MFI; EORTC QLQ-C30	Scoping review	Goncalves Leite Rocco et al.	2024
Cancer	FACIT-F; PedsQL	Systematic review	Hao et al.	2023
Cancer	EORTC QLQ-C30	Interventional study	Ibrahim et al.	2024
Cancer	BFI	Retrospective cohort study	Mack et al.	2021
Cancer	CFQ	Interventional study	Nilsson et al.	2020
Cancer	MFI; EORTC QLQ-C30; RPS; FACIT-F; FACT-F; LASA; FAS; FAQ; PROMIS	Systematic review	Penna et al.	2023
Cancer	PipFS-R; MFI; FACT-F; BFI; FACIT-F	Interventional study	Trommer et al.	2023
Cancer	MFI; EORTC QLQ-C30; FQ; VAS; BFI	Systematic review	Vira et al.	2021
Cancer (breast and prostate)	PipFS; FACIT-F; FACT-F; MFI; FAQ	Systematic review	Cano-Uceda et al.	2025
Cancer (breast and prostate)	MFI	Randomized trial	Van de Wiel et al.	2021
Cancer (breast)	FSS; EORTC QLQ-C30	Case–control study	Alaca et al.	2024
Cancer (breast)	FIS	Interventional study	Bahçaci et al.	2024
Cancer (breast)	BFI; EORTC QLQ-C30	Pilot study	Invernizzi et al.	2020
Cancer (breast)	PipFS	Randomized trial	Mur-Gimeno et al.	2024
Cancer (breast)	MFI-SF ; PipFS	Systematic review	Mur-Gimeno et al.	2022
Cancer (breast)	EORTC QLQ-C30	Randomized trial	Rashid et al.	2025
Cancer (breast)	BFI	Pilot study	Tay et al.	2024
Cancer (breast)	PipFS	Systematic review	Wang et al.	2022
Cancer (colorectal)	EORTC QLQ-C30	Interventional study	Finch et al.	2024
Cancer (gliomas)	HRQoL	Randomized trial	Hansen et al.	2020
Cancer (gynecologic)	Not specified	Case report	Coughenour et al.	2024
Cancer (gynecologic)	MFIS	Interventional study	Wood et al.	2022
Cancer (head and neck)	EORTC QLQ-C30	Interventional study	Felser et al.	2025
Cancer (head and neck)	FSS	Survey	Krishna Alaparthi et al.	2022
Cancer (head and neck)	FACT-F	Randomized trial	McNeely et al.	2024
Cancer (hematologic)	FACIT-F	Randomized trial	Accogli et al.	2022
Cancer (lung with spinal cord injury)	FACIT-F	Case report	Kelch et al.	2022
Cancer (lymphoma)	Not specified	Systematic review	Jerbi et al.	2022
Cancer (myeloma)	FACIT-F	Randomized trial	McCourt et al.	2023
Cancer (esophageal)	MFI; EORTC QLQ-C30	Randomized trial	Van Vulpen et al.	2021
Cancer (esophagogastric)	Not specified	Qualitative study	O'Neill et al.	2021
Cancer (pediatrics acute lymphoblastic leukemia)	PedsQL	Randomized trial	Elnaggar et al.	2025
Cancer (pediatrics and adolescents)	Not specified	Cross-sectional survey	Ospina et al.	2020
Cancer (pediatrics and adolescents)	PedsQL; MFS; CCFS; FS-C; FS-A	Literature review	Ospina et al.	2021
Cancer (survivor)	FACIT-F; PipFS-R	Systematic review	Gray et al.	2024
Cancer (urinary bladder)	PipFS	Randomized trial	Porserud et al.	2024
Cerebral Palsy (children)	PedsQL	Interventional study	Celikel et al.	2023
Charcot–Marie–Tooth disease	FSS; CIS-20R	Scoping review	Tedeschi et al.	2025
Chronic advanced diseases (palliative)	3LNQ	Cross-sectional study	Høgdal et al.	2020
Chronic back pain	Not specified	Pilot study	You et al.	2021
Chronic low back pain	FACIT-F	Randomized trial	Dilekçi et al.	2020
Chronic obstructive pulmonary disease	FSS	Randomized trial	Kovelis et al.	2020
Chronic widespread pain	CFQ	Interventional study	Thompson et al.	2022
Coronary artery bypass graft	Not specified	Survey	Hong et al.	2020
Coronary artery disease	MBS	Interventional study	Saklıca et al.	2024
COVID-19	MBS; Quality of life (not specified)	Systematic review	Beqaj et al.	2022
COVID-19	MBS	Randomized trial	Fereydounnia et al.	2022
COVID-19	FSS	Randomized trial	Paneroni et al.	2024
COVID-19	VAS	Systematic review	A.M.C. et al.	2023
COVID-19 (long)	FSS	Pilot study	Deodato et al.	2024
COVID-19 (long)	mMRC; FSS	Systematic review	Mass Kokolevich et al.	2024
COVID-19 (long)	CIS-Fatigue	Randomized trial	Volckaerts et al.	2023
COVID-19 (post)	VAS	Interventional study	Tăbîrță et al.	2023
COVID-19 (post)	MPFSDQ	Controlled open study	Campos et al.	2024
COVID-19 (post)	Not specified	Systematic review	Dillen et al.	2023
COVID-19 (post)	Fatigue outcomes were limited and could not be synthesized	Systematic review	Pouliopoulou et al.	2023
COVID-19 (post)	MBS	Randomized trial	Şahın et al.	2023
COVID-19 (post)	FSS	Pilot study	Sarmento et al.	2024
COVID-19 (post)	BRS; CFS-11; VAS; mMRC	Systematic review	Valverde-Martínez et al.	2023
COVID-19 (post)	FSS	Observational study	Weigl et al.	2024
COVID-19 pneumonia (severe)	FSS; CFQ; FACIT-F	Interventional study	Asimakos et al.	2023
Fibromyalgia	PipFS-R	Randomized trial	Fonseca et al.	2021
Fibromyalgia	MAF	Randomized trial	Yoo et al.	2022
Functional neurological disorder	PROMIS	Case series	Mowry et al.	2025
Guillain–Barré syndrome	FSS	Survey	Davidson et al.	2022
Guillain–Barré syndrome	FSS	Randomized trial	Shah et al.	2022
Healthy male	Self-reported perceived muscle fatigue	Experimental interventional study	Lim et al.	2024
Healthy workers	Not specified	Experimental interventional study	Vitoulas et al.	2022
Hematological diseases	Not specified	Interventional study	Morais et al.	2023
Hereditary cerebellar ataxia	FSS	Randomized trial	Milne et al.	2025
Hereditary neuromuscular diseases	FSS	Cross-sectional study	Andries et al.	2022
Hereditary neuromuscular diseases	Not specified	Survey	Stępień et al.	2022
Hip fracture surgery	VRS	Randomized trial	Zilmer et al.	2024
Hypermobility spectrum disorder	CIS-subscale fatigue	Feasibility study	Liaghat et al.	2020
Inflammatory arthritis	NRS	Randomized trial	Nordén et al.	2024
Interstitial lung disease	FACIT-F	Case report	Dasouki et al.	2023
Juvenile myasthenia gravis	PedsQL	Randomized trial	Mohamed et al.	2022
Long-term musculoskeletal conditions	NRS	Feasibility study	Minns Lowe et al.	2020
Marfan syndrome	FSS	Case report	Bhagwatkar et al.	2024
Multiple sclerosis	MFIS; WEIMuS; FIS; FSI; FSS	Scoping review	Adnan et al.	2024
Multiple sclerosis	MFIS	Systematic review	Amedoro et al.	2020
Multiple sclerosis	FIS	Interventional study	Andrejeva et al.	2023
Multiple sclerosis	FSS; MBS	Randomized trial	Blázquez-Fernández et al.	2024
Multiple sclerosis	FSS; MFIS	Randomized trial	Bonnyaud et al.	2025
Multiple sclerosis	MFIS	Pilot study	Chanpimol et al.	2020
Multiple sclerosis	MFIS	Interventional study	Dastan et al.	2025
Multiple sclerosis	FSS	Interventional study	Drużbicki et al.	2021
Multiple sclerosis	MFIS	Systematic review	Elhusein et al.	2024
Multiple sclerosis	WEIMuS	Randomized trial	Flachenecker et al.	2020
Multiple sclerosis	FSS	Interventional study	Ghosh et al.	2024
Multiple sclerosis	FSS	Interventional study	Hendricksen et al.	2024
Multiple sclerosis	MFIS	Interventional study	Hrušková et al.	2024
Multiple sclerosis	MFIS	Randomized trial	Karakas et al.	2025
Multiple sclerosis	FSS	Interventional study	Knapova et al.	2025
Multiple sclerosis	MFIS	Interventional study	Kumar et al.	2024
Multiple sclerosis	FSS; MFIS	Randomized trial	Lamberti et al.	2020
Multiple sclerosis	FAS	Randomized trial	Lysogorskaia et al.	2023
Multiple sclerosis	MSQOL-54; MFIS; WEIMuS; FIS; FSS	Systematic review	Najafi et al.	2025
Multiple sclerosis	MFIS	Pilot study	Ozdogar et al.	2022
Multiple sclerosis	FSS	Randomized trial	Ozsoy-Unubol et al.	2022
Multiple sclerosis	FSS	Interventional study	Petracca et al.	2024
Multiple sclerosis	FSS; MFIS; CIS-20R; FSMC	Systematic review	Taul-Madsen et al.	2021
Multiple sclerosis	FSS	Observational study	Torchio et al.	2025
Multiple sclerosis	FIS; FSMC; FSS; MFIS; PROMIS; WEIMuS; RFT	Systematic review	Torres-Costoso et al.	2022
Multiple sclerosis	FSS	Randomized trial	Yazgan et al.	2020
Multiple sclerosis	FSS; FIS	Randomized trial	Yucekaya et al.	2025
Multiple sclerosis (pediatric onset)	FSS	Interventional study	Vural et al.	2023
Multiple sclerosis with Parkinson disease	FIS	Case report	Sütçü et al.	2021
Myalgic encephalomyelitis/chronic fatigue syndrome	CFQ; CIS; FSS; PROMs	Systematic review	Wormgoor et al.	2021
Myalgic encephalomyelitis/chronic fatigue syndrome (adolescents)	Self-reported	Randomized trial	Anderson et al.	2020
Myotonic dystrophy type 1	PROMIS	Single subject experimental design	Fossmo et al.	2024
Older adults	FSS	Randomized trial	Garbin et al.	2024
Parkinson disease	ParkFS	Feasibility study	Cooley Hidecker et al.	2022
Parkinson disease	ParkFS; MFIS; VAS; FSS; PipFS-R; KFSS	Systematic review	Ernst et al.	2023
Pediatric hematopoietic stem cell transplant	PROMIS	Randomized trial	Smith et al.	2022
People with disabilities	Not specified	Randomized trial	Ravesloot et al.	2022
Persisting post-concussive Symptoms	FSS	Randomized trial	Mercier et al.	2025
Pituitary adenoma	FSS	Randomized trial	Dülger et al.	2022
Postconcussion symptoms	BFI	Randomized trial	Nguyen et al.	2023
Primary biliary cholangitis	PBC-40 fatigue domain	Clinical trial	Freer et al.	2024
Pulmonary hypertension	FSS	Systematic review	Luo et al.	2022
Refractory dyspnea	CRQ	Pilot study	Clark et al.	2025
Spinal cord injury	FAS	Feasibility study	Postol et al.	2021
Stroke (early)	Dyspnea fatigue score	Systematic review	Zhang et al.	2024
Stroke (minor) and transient ischemic attack	FSS	Randomized trial	Deijle et al.	2022
Stroke (subacute, mild to moderate)	MFI	Feasibility study	Clague-Baker et al.	2022
Systemic sclerosis	NRS	Randomized trial	Yakut et al.	2021
Transverse myelitis	FAS	Case report	Choubisa et al.	2022

3LNQ, 3-Levels-of-Needs Questionnaire; BFI, Brief Fatigue Inventory; BRS, Borg Rating Scale; CCFS, Childhood Cancer Fatigue Scale; CFS-11, Chalder Fatigue Score; CFQ, Chalder Fatigue Questionnaire/Scale; CIS, Checklist of Individual Strength; CIS-20R, Checklist Individual Strength-Revised; CRQ, Chronic Respiratory Questionnaire; EORTC QLQ-C-30, European Organisation for the Research and Treatment of Cancer Quality of Life Questionnaire Core-30; FACIT-F; Functional Assessment of Chronic Illness Therapy - Fatigue Scale; FACT-F, Functional Assessment of Cancer Therapy-Fatigue Scale; FAQ, Fatigue Assessment Questionnaire; FAS, Fatigue Assessment Scale; FIS, Fatigue Impact Scale; FQ, Fatigue Questionnaire; FS-A, Fatigue Scale for adolescents; FS-C, Fatigue Scale for a child; FSI, Fatigue Symptom Inventory; FSMC, Fatigue Scale for Motor and Cognitive functions; FSS, Fatigue Severity Scale; HRQoL, Health-Related Quality of Life; KFSS, Krupp Fatigue Severity Scale; LASA, Linear Analog Scale Assessment; MAF, Multidimensional Assessment of Fatigue; MBS, Modified Borg Scale; MFI, Multidimensional Fatigue Inventory; MFIS, Modified Fatigue Impact Scale; MFI-SF, Multidimensional Fatigue Inventory Short Form; MFS, Multidimensional Fatigue Scale; mMRC, Modified British Medical Research Council; MPFSDQ, Modified Pulmonary Functional Status and Dyspnea Questionnaire; MSQOL-54, Multiple Sclerosis Quality of Life-54; NFI-MND, Neurological Fatigue Index-motor neuron disease; NRS, Numerical Rating Scale; ParkFS, Parkinson’s Fatigue Scale; PBC-40, primary biliary cholangitis-40 fatigue domain; PedsQL, Pediatric Quality of Life Inventory-Multidimensional Fatigue Scale; PipFS, Piper Fatigue Scale; PipFS-R, Revised Piper Fatigue Scale; PROMIS, Patient-Reported Outcomes Measurement Information System; PROMs, Patient-Reported Outcomes Measures; RFT, Rotten fatigue test; RPS, Revised Piper Fatigue Scale; VAS, Visual Analogue Scale; VRS, Verbal Rating Scale; WEIMuS, the Würzburg Fatigue Inventory for Multiple Sclerosis

## Results

A total of 139 articles met the criteria for our research^12–150)^. We found 43 different outcomes (distinguishing modified versions for certain scales) and 46 different population groups, which we categorized into 6 distinct fields: neurology and neuromuscular; cancer (all types); pneumology (including coronavirus disease 2019 [COVID-19]); musculoskeletal; cardiovascular; other fields (rare disease, healthy population, etc.).

**Fig. 3.**
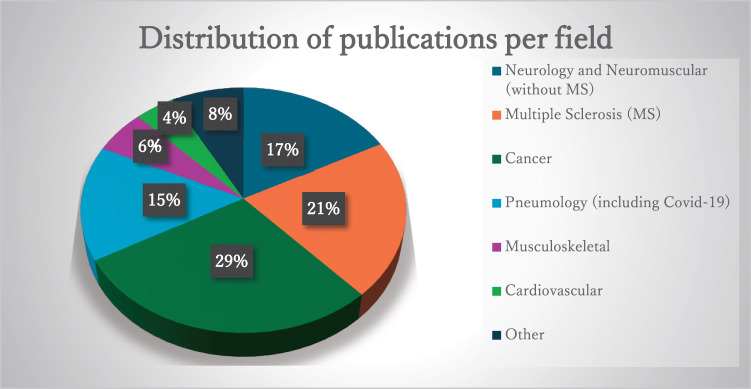
Distribution ratio of publications per field. COVID-19, coronavirus disease 2019; MS, multiple sclerosis

The number of publications per field was as follows: 40 publications for cancer (29%); 29 on multiple sclerosis (MS) (21%) and 24 on other pathologies (17%) for neurology and neuromuscular; 21 publications including 16 on COVID-19 for Pneumology (15%); 8 publications for musculoskeletal (6%); 6 publications for cardiovascular (4%); and 11 publications for other fields (8%) (Fig. 3).

**Fig. 4.**
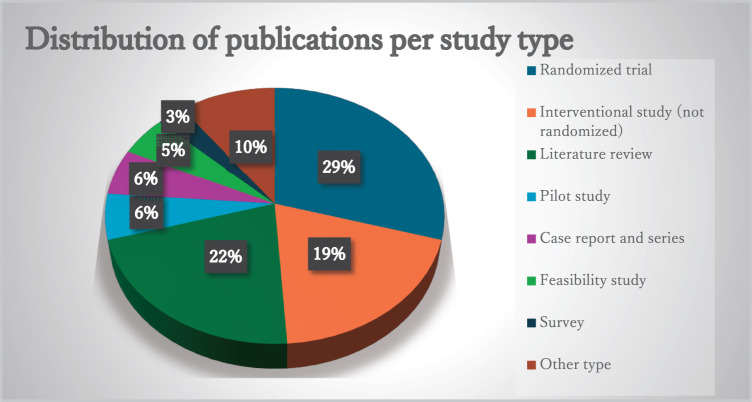
Distribution ratio of publications per study type.

The number of publications per study type was as follows: 41 publications for randomized trial (29%); 30 publications including 27 systematic review and 3 scoping review for literature review (22%); 27 publications for interventional study (19%); 8 publications for pilot study (6%); 8 publications for case report and case series (6%); 7 publications for feasibility study (5%); 4 publications for survey (3%); and 14 publications for other types (10%) (Fig. 4).

Since our research period included lockdown periods, 23 articles used or mentioned remote intervention techniques (e.g., tele-rehabilitation or tele-physiotherapy)^14,19,21,27,28,39–41,51,56,72,81,99,117,121,122,129–132,136,142,148)^.

**Fig. 5.**
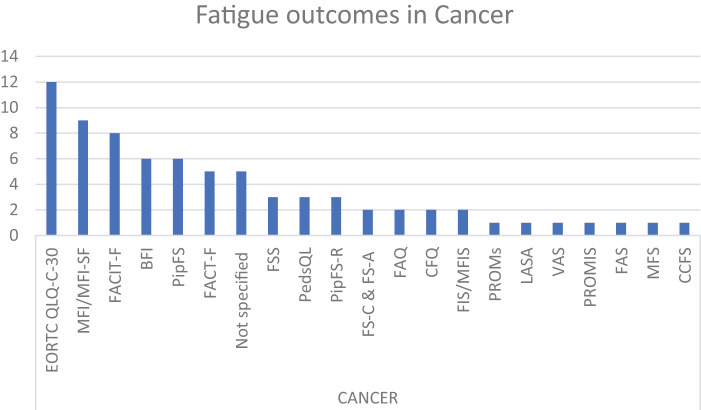
Number of fatigue outcomes found in cancer. BFI, Brief Fatigue Inventory; CCFS, Childhood Cancer Fatigue Scale; CFQ, Chalder Fatigue Questionnaire/Scale; EORTC QLQ-C-30, European Organisation for the Research and Treatment of Cancer Quality of Life Questionnaire Core-30; FACIT-F; Functional Assessment of Chronic Illness Therapy - Fatigue Scale; FACT-F, Functional Assessment of Cancer Therapy-Fatigue Scale; FAQ, Fatigue Assessment Questionnaire; FAS, Fatigue Assessment Scale; FIS, Fatigue Impact Scale; FS-A, Fatigue Scale for adolescents; FS-C, Fatigue Scale for a child; FSS, Fatigue Severity Scale; LASA, Linear Analog Scale Assessment; MFI, Multidimensional Fatigue Inventory; MFIS, Modified Fatigue Impact Scale; MFI-SF, Multidimensional Fatigue Inventory Short Form; MFS, Multidimensional Fatigue Scale; PedsQL, Pediatric Quality of Life Inventory-Multidimensional Fatigue Scale; PipFS, Piper Fatigue Scale; PipFS-R, Revised Piper fatigue scale; PROMIS, Patient-Reported Outcomes Measurement Information System; PROMs, Patient-Reported Outcomes Measures; VAS, Visual Analogue Scale

**Fig. 6.**
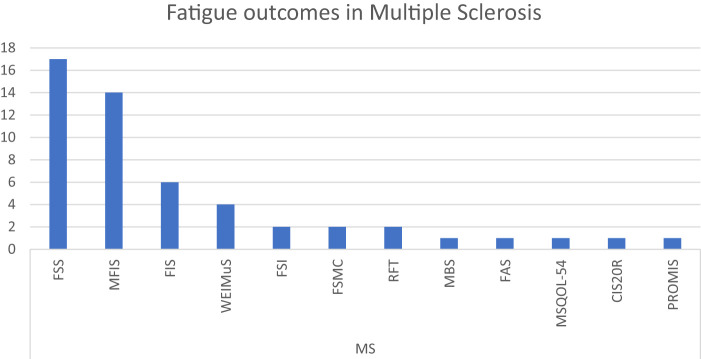
Number of fatigue outcomes found in multiple sclerosis. CIS-20R, Checklist Individual Strength-Revised; FAS, Fatigue Assessment Scale; FIS, Fatigue Impact Scale; FSI, Fatigue Symptom Inventory; FSMC, Fatigue Scale for Motor and Cognitive functions; FSS, Fatigue Severity Scale; MBS, Modified Borg Scale; MFIS, Modified Fatigue Impact Scale; MS, mutiple sclerosis; MSQOL-54, Multiple Sclerosis Quality of Life-54; PROMIS, Patient-Reported Outcomes Measurement Information System; RFT, Rotten fatigue test; WEIMuS, The Würzburg Fatigue Inventory for Multiple Sclerosis

**Fig. 7.**
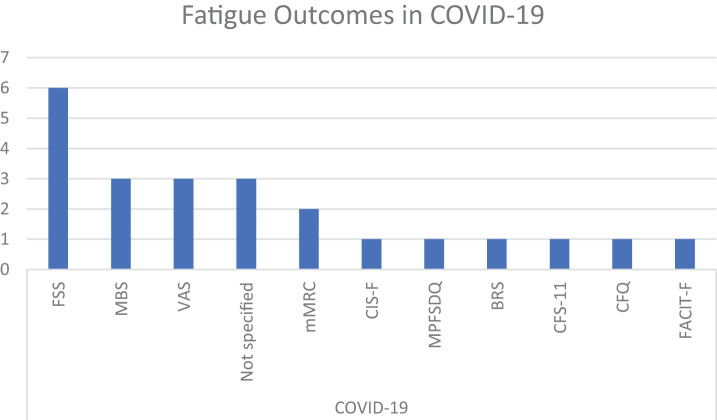
Number of fatigue outcomes found in COVID-19. BRS, Borg Rating Scale; CFQ, Chalder Fatigue Questionnaire/Scale; CFS-11, Chalder Fatigue Score; CIS-F, fatigue severity subscale of the Checklist of Individual Strength; COVID-19, coronavirus disease 2019; FACIT-F, Functional Assessment of Chronic Illness Therapy – Fatigue Scale; FSS, Fatigue Severity Scale; MBS, Modified Borg Scale; mMRC, Modified British Medical Research Council; VAS, Visual Analogue Scale; MPFSDQ, Modified Pulmonary Functional Status and Dyspnea Questionnaire

Unsurprisingly, and in accordance with the literature, the 3 most commonly represented diseases were studies involving patients with cancer, MS, and post-COVID-19^151,152)^. For the latter, we investigated further what different outcomes were used and whether some of them were more representative (Figs. 5–7).

In cancer, we found 21 different outcomes across the 40 publications^20,23,25,31,33,34,47,48,53,56,66,69,70,72,74,75,80,82,84,86,87,93,94,102.105,109,110,116,120,122,123,124,126,128,130,133,144,147)^. In one of the articles, although it is a similar scale, the authors distinguish between the fatigue scale for children and adolescents. The most representative scales are the European Organisation for the Research and Treatment of Cancer Quality of Life Questionnaire Core-30(EORTC QLQ-C-30; 12 articles), Multidimensional Fatigue Inventory (MFI; 8 articles), Functional Assessment of Chronic Illness Therapy – Fatigue Scale (FACIT-F; 8 articles), and 8 articles including the Piper Fatigue Scale (PipFS-R) and Revised Piper Fatigue Scale (RPS). We also noted that the Piper Fatigue Scale, designed to assess fatigue in women with breast cancer, was used in only 4 of the 10 articles dealing with this topic.

In MS, we found 12 different outcomes across the 29 publications^14,19,21,24,27,37,39–41,46,50,62,63,65,67,71,85,89,91,92,97,100,129,131,135,138,139,143,150)^. The most representative scale was the Fatigue Severity Scale (FSS; 17 articles), followed by the Modified Fatigue Impact Scale and its original version (17 articles including 14 MFIS and 6 Fatigue Impact Scale [FIS]). Although certain fatigue scales are appropriate for the MS population (notably the FIS, MFIS, FSS, and the Würzburg Fatigue Inventory for Multiple Sclerosis [WEIMuS]), we have noted that there is disparity in their use.

In COVID-19, we found 11 different outcomes across the 16 publications^16,17,22,51,59,68,81,95,111,115,118,119,132,134,142,148)^. The most representative scale was FSS (6 articles), followed by the Modified Borg Scale (MBS; 3 articles) and Visual Analogue Scale (VAS; 3 articles). The choice of these scales appears to be a consequence of the pulmonary involvement and the context in which the assessments were conducted.

We also noted that some literature reviews included both unidimensional and multidimensional outcomes of fatigue, some of which addressed different time periods of symptoms experienced by patients (at the time, since a week, since last 4 weeks, etc.)^19,29,33,47,66,69,75,92,96,101,103,107,108,110,116,119,124,128,129,132,141,142,144)^.

## Discussion

A total of 139 articles published between 2020 and mid-July 2025 met the eligibility criteria for this scoping review. To our knowledge, this is the 1st exploratory study to examine fatigue assessments performed by physical therapists. Although the COVID-19 pandemic is likely influencing our interest in fatigue in the field of rehabilitation, it appears that these topics have always been studied in light of our initial findings during the screening phase (Fig. 1). Although our research was not intended to describe every scale of fatigue, the variety of different outcomes appeared to us to be very large. This initial observation leads us to a notion that is not new but is nevertheless evident in practice: fatigue is a multidimensional and complex phenomenon that is challenging to assess.

In the research field, some instrumental methods can be used to objectivize certain dimensions of fatigue through performance tests, such as neuromuscular or cognitive fatigability^153,154)^. However, these measures are limited by the experimental conditions, conducted in controlled environments, which do not necessarily reflect the nature and experience of fatigue in subjects’ everyday lives. This perception, known as subjective fatigue, is assessed in most cases by a general or specific questionnaire, based on the respondent’s self-estimation. However, if a primary impairment (pathology) can modify an individual’s perception of fatigue, it would seem difficult to consider its dominance over other influencing factors. This is probably why many outcomes have been developed and continue to be revised and modified, as the symptoms are not always clinically describable. Several theories indicate that the sensation of fatigue must be associated with the anatomical brain structures responsible for conscious perception, rather than with a single peripheral physiological disturbance or altered metabolic state^155)^.

According to Joseph Levine’s theory, there could be an explanatory gap in the fatigue perceived by an individual before and after a change in their health status^156)^. Knowing an individual’s baseline level of fatigue perception could be a 1st step toward understanding which dimensions are most affected in an individual after illness. Conversely, in conditions affecting individuals from an early age (hereditary diseases, cerebral palsy, etc.), it is difficult to distinguish the role played by the disease in the perception of fatigue that children develop as soon as they become conscious. In other words, why do children with the same congenital condition not perceive or express fatigue in the same manner?

This consideration leads us to consider other factors that can significantly influence the perception of fatigue. Certain parameters have already been mentioned in various studies, such as anthropometric factors (gender, age), sociocultural factors, and professional factors^157–159)^. These intra- and interpersonal experiences could contribute to the development of a representation of fatigue that is specific to each individual and in each dimension. However, this does not diminish the importance of assessing fatigue in patients who are limited in their daily activities and whose quality of life is impaired by this almost invisible, but clearly present symptom.

Due to its frequent occurrence in many chronic diseases, fatigue assessment has become essential in rehabilitation interventions. As we have seen in our study, this appears to be a significant outcome in protocols involving physical therapists. However, few studies argue for the choice of scale used to measure fatigue, nor do they hypothesize about which specific dimension or aspect of fatigue their intervention might improve. Positive results are reported in several studies, but they appear difficult to reproduce.

At the same time, its assessment appears less standardized in other areas such as musculoskeletal disorders. While physiotherapy can have beneficial effects on motor performance, it would seem a shame not to investigate further the subjective fatigue of individuals to better understand the relationship between performance and perception.

Finally, we have no explicit arguments regarding the disparity of the scales used. While some scales have been specifically developed for a particular pathology, some authors use generic scales in their work. However, we put forward 3 hypotheses that could explain this finding in line with the literature found^160)^. The 1st is that the scales are not available or accessible. This may be the case when the use of assessments requires training or when they are not validated in the required language. The 2nd hypothesis is the field context. Some scales are longer or more complex to implement within the department. Due to time or resource constraints, the choice of scales may be reduced to the assessment of a single dimension or the shortest one to be administered. Finally, the last hypothesis relates to the design of the protocol. The physical therapist may be the evaluator in a trial with a predefined protocol. In this case, the choice of scale is predetermined and cannot be changed. On this last point, we considered the possibility that some protocols may choose, rightly or wrongly, a fatigue scale simply because it was designed for the target population. However, in light of all our comments above, we suggest that the scale chosen should not only reflect the specific characteristics of the population but also the variable of fatigue on which the intervention is supposed to be effective.

The key points that emerge from our exploratory study are as follows:

- Fatigue assessed in rehabilitation must be better defined in the objectives to choose the appropriate outcome for the type of intervention.
- A common guide for clinicians and researchers to choose the most appropriate fatigue measurement scale could contribute to better reproducibility of studies in the future.
- Accessible materials and training would help physical therapists to use the appropriate scales.
- A more standardized assessment of subjective fatigue in minority fields would contribute to a better understanding of the effects of physical therapy on fatigue in general.
- Due to the multidimensional nature of fatigue, research in this field should involve both other areas of rehabilitation and various scientific disciplines.
- Develop an assessment method that also considers an individual’s representations of their own fatigue with a qualitative approach^161)^.

This comprehensive rapid scoping review has provided an initial overview of the various outcomes used in physical therapy and in different fields. We hope that this document will be a useful resource for improving the care of patients undergoing rehabilitation. Future work could build on these observations to develop recommendations and conduct studies using a more comprehensive fatigue assessment. Given the large number of potential articles in other areas of rehabilitation on this topic, a more extensive literature review would also be useful for the next stage.

## Limitations

This scoping review of the literature is time-bound: articles published before 2020 were not included. Articles without results (protocol design), posters, or conference presentations were also not included. Although fatigue is a phenomenon studied worldwide in physiotherapy, only articles in English were included in our research. Due to the exploratory nature of the study and the fact that we limited our research to physiotherapy interventions, our findings do not provide a comprehensive overview of fatigue assessments used in the rehabilitation field in general. Not all of the recommendations for conducting a scoping review could be met. In particular, the participation of a librarian would have helped to complete the research strategy for an exhaustive search on our topic^7)^.

## Supplementary Materials

Appendix 1: Preferred reporting items for systematic reviews and meta-analyses extension for scoping reviews (PRISMA-ScR) checklist.

Appendix 2: Search string strategies for each database.
