# Supplementary material for: Take Fatigue or Fatigues into Account in Physiotherapy Interventions? A Rapid Scoping Review
Source: Phys Ther Res. 2025 Nov 15;28(3):157–73. doi: 10.1298/ptr.R0038 (PMC12778359; doi:10.1298/ptr.R0038)
Supplement: Supplementary file 2 — Appendix 2: Search string strategies for each database. [file ptr-28-157-s002.pdf]

Appendix 2: Search string strategies for each database

PubMed:

(("physical therapists"[MeSH Terms] OR "physical therap*"[Title/Abstract] OR "physiotherap*"[Title/Abstract]) AND ("rehabilitation"[MeSH Terms] OR "rehabilitation*"[Title/Abstract] OR "habilitation*"[Title/Abstract]) AND ("evaluation*"[Title/Abstract] OR "assessment*"[Title/Abstract] OR "measure*"[Title/Abstract]) AND ("fatigue"[MeSH Terms] OR "fatigue*"[Title/Abstract] OR "tiredness*"[Title/Abstract] OR "weariness*"[Title/Abstract] OR "lassitude*"[Title/Abstract] OR "weakness*"[Title/Abstract])) AND ((y_10[Filter]) AND (excludepreprints[Filter]) AND (english[Filter]))

Cochrane Library:

#1 ("physical therapist"):ti,ab,kw 1689

#2 physiotherap* 31456

#3 physical therap* 96434

#4 #1 OR #2 OR #3 114969

#5 MeSH descriptor: [Rehabilitation] explode all trees 56429

#6 habilitation* 179

#7 rehabilitation* 92487

#8 #5 OR #6 OR #7 128313

#9 ("evaluation"):ti,ab,kw 248551

#10 evaluation* 283522

#11 assessment* 394695

#12 measure* 614149

#13 #9 OR #10 OR #11 OR #12 984650

#14 MeSH descriptor: [Fatigue] explode all trees 6109

#15 fatig* 52298

#16 tiredness* 1988

#17 weariness* 0

#18 lassitude* 231

#19 weakness* 11560

#20 #14 OR #15 OR #16 OR #17 OR #18 OR #19 62723

#21 #4 AND #8 AND #13 AND #20 3331

Web of Science:

1 Search: TS= (physical therap* OR physiotherap*) Results: 193304

2 Search: TS= (rehabilitation* OR habilitation*) Results: 321678

3 Search: TS= (fatig* OR tiredness* OR weariness* OR lassitude* OR weakness* NOT burn out) Results: 536755

4 Search: TS= (evaluation* OR assessment* OR measure*) Results: 13258925

5 Search #1 AND #2 AND #3 AND #4 and 2025 or 2010 or 2011 or 2012 or 2013 or 2014 or 2015 or 2016 or 2017 or 2019 or 2020 or 2018 or 2021 or 2022 or 2023 or 2024 (Final Publication Year) and 2014 or 2013 or 2012 or 2011 or 2010 (Exclude – Final Publication Year) Results: 1148

CINHAL (EBSCO):

AB (fatig* OR tiredness* OR weariness* OR lassitude* OR weakness*) AND AB (evaluation* OR assessment* OR measure*) AND AB (rehabilitation* OR habilitation*) AND AB (physical therap* OR physiotherap*)

Embase :

1 (physical AND therap* OR physiotherap* OR physical) AND ('therapist'/exp OR therapist) OR 'physiotherapist'/exp OR physiotherapist
Results: 52,339

2 rehabilitation* OR habilitation* OR 'rehabilitation'/exp OR rehabilitation
evaluation* OR assessment* OR measure*
Results: 1,219,124

3 fatig* OR tiredness* OR weariness* OR lassitude* OR weakness* OR 'fatigue'/exp OR fatigue
Results: 11,303,374

4 #1 AND #2 AND #3 AND #4
Results: 660,708

5 #5 AND (2015:py OR 2016:py OR 2017:py OR 2018:py OR 2019:py OR 2020:py OR 2021:py OR 2022:py OR 2023:py OR 2024:py OR 2025:py) Results: 1339

LiSSA:

(((((((kinésithérapeutes.tl OU kinésithérapeutes.mc) OU kiné*.tl) OU kiné*.mc) OU masseur kiné*.tl) OU masseur kiné*.mc) OU physiothérap*.tl) OU physiothérap*.mc) ET (((((((rééducation*.tl OU rééducation*.mc) OU Réadaptation.tl) OU Réadaptation.mc) OU médecine physique et de réadaptation.tl) OU médecine physique et de réadaptation.mc) OU réadapt*.tl) OU réadapt*.mc) ET (((((évaluation*.tl OU évaluation*.mc) OU mesure*.tl) OU mesure*.mc) OU appréciation*.tl) OU appréciation*.mc) ET (((((((fatigue.tl OU fatigue.mc) OU fatig*.tl) OU fatig*.mc) OU lassitud*.tl) OU lassitud*.mc) OU épuisement*.tl) OU épuisement*.mc)
